# Plating for Intra-articular Fractures of the Distal Femur: Functional and Radiological Outcomes

**DOI:** 10.7759/cureus.33207

**Published:** 2023-01-01

**Authors:** Hrushikesh Bandaru, Arun H Shanthappa

**Affiliations:** 1 Orthopaedics, Sri Devraj Urs Medical College, Kolar, IND

**Keywords:** neer’s score, locking compression plate, radiological outcome, functional outcome, distal femoral fracture

## Abstract

Background and objective

Intra-articular fractures of the distal femur pose a significant surgical difficulty. These fractures are challenging to repair, and surgery is frequently advised for a successful outcome. The distal femoral fractures make up between 4-6% of all femoral fractures and account for less than 1% of all fractures. When compared to the single screw's axial stiffness or pullout resistance, as is the case with unlocked plates, the locking compression plate (LCP) is a single beam construct whose strength of fixation is equal to the sum of all screw-bone contacts. Against this backdrop, the current study was conducted for evaluating the functional outcomes of distal femoral fractures treated with LCP fixation.

Methodology

We conducted an observational study at a tertiary care facility in Kolar spanning a period of three years, from January 2019 to January 2022. We included all patients aged more than 18 years, who were diagnosed with distal femoral fractures [only those classified as type C according to the AO Foundation/Orthopedic Trauma Association (AO/OTA) classification]. Patients with terminal illnesses, revision of previous surgery, fractures with neurovascular injuries, and those diagnosed with pathological fractures were excluded from the study. The included patients were treated by LCP and discharged on postoperative day 10 after suture removal. The first follow-up was at the first month and subsequent follow-ups were done at three and six months, and the functional outcomes were assessed by using Neer’s score for straight leg raises. Radiographs with fading fracture lines and callus formation on three-fourths of the cortices were regarded as indicators of fracture healing.

Results

Among the 30 study participants, 80% were males. The mean time for the radiological union was 15 weeks. In this study, there were no instances of infection or angular deformity greater than 5 degrees. About 80% of the study participants had ranges of motion (ROM) above 120 degrees at the end of the six-month follow-up period; 12 cases had an excellent score (40%), 15 cases (50%) had a satisfactory score, two cases (6.7%) had an unsatisfactory score, and only one case had poor score. Common complications observed were excessive bleeding, difficulty in reduction, superficial infections, and knee stiffness.

Conclusion

For patients with distal femoral fractures, the LCP treatment can result in good functional and radiological outcomes with fewer complications.

## Introduction

Distal femur intra-articular fractures pose a significant surgical difficulty. These fractures are challenging to repair, and surgery is frequently advised for a successful outcome. These are typically comminuted and intra-articular, and they are linked to high-energy trauma in young people and osteoporotic bones in the elderly [1-3].

According to reports, distal femoral fractures make up between 4-6% of all femoral fractures and account for less than 1% of all fractures [4]. Normal causes of supracondylar femur fractures include two distinct methods of injury and two distinct populations. Firstly, in young adults following high-energy trauma (60% of these are males under 40 years of age; injuries sustained in sports and accidents), and then in the elderly following low-energy trauma (60% of these are females older than 60 years of age; falls, sprains, etc.) [5-7].

Fractures at the distal end of the femur need to be operated on for better results. The traditional surgical treatment options include plating (blade plating, dynamic condylar screws), retrograde and antegrade nailing, and external fixation. The use of locking plate fixation for complicated distal femur fractures has been on the rise. When compared to the single screw's axial stiffness or pullout resistance, as is the case with unlocked plates, the locking compression plate (LCP) is a single beam construct whose strength of fixation is equal to the sum of all screw-bone contacts [8]. The tendency for varus collapse, which can be a problem with conventional lateral plates, is reduced by the distal femoral locking compression plate (DFLCP), which provides multiple points of fixed-angle contact between the plate and screws in the distal end of the femur even with a small epiphyseal segment [9].

The current generation of DFLCPs comprises fixed-angle constructs that have been pre-contoured based on the typical bony anatomy of adults. Locking screws are more useful in osteoporotic bones than conventional screws because of their higher pullout strength. These plates are meant to be applied in a minimally invasive manner to protect local biology, prevent issues with fracture healing, and prevent infection [10,11].

Against this background, the current study focused on evaluating the functional and radiological outcomes in patients with distal femoral fractures treated with LCP fixation.

## Materials and methods

Study design and setting

This was an observational study conducted at a tertiary care facility in Kolar, covering a period of three years from January 2019 to January 2022.

Eligibility criteria

Inclusion Criteria

We included all patients aged more than 18 years, who were diagnosed with distal femoral fractures and were admitted to our hospital during the study period. Only those patients with type C distal femoral fractures according to the AO Foundation/Orthopedic Trauma Association (AO/OTA) classification were included in the study.

Exclusion Criteria

Patients with terminal illnesses and those diagnosed with pathological fractures were excluded from the study. These patients were treated with LCP which was fixed.

Sampling technique and sample size

The rate of favorable functional outcome of the LCP was 92%, according to Agrawal et al. [12]. This finding was based on a prospective study involving 12 patients who had distal femur fractures and were treated with LCP. Using the formula 3.84*p*q/d2, where p is prevalence, q is the complement of p, and d is absolute precision, it was determined that the study required a minimum sample size of 30 with a 10% absolute error and a 95% confidence interval.

Data collection

These patients were treated by LCP and discharged on postoperative day 10 after suture removal. The first follow-up was at the first month and subsequent follow-ups were done at three months and six months, and the functional outcomes were assessed by using Neer’s score for straight leg raises. Radiographs with fading fracture lines and callus formation on three-fourths of the cortices were regarded as indicators of fracture healing.

Data entry and analysis

The data were entered into Microsoft Excel. The results were analyzed using IBM SPSS Statistics version 21 (IBM Corp., Armonk, NY). All categorical data were expressed as percentages and the continuous variables were expressed as mean with standard deviation.

## Results

A total of 30 participants were included in the current study. The mean age of the study participants was 48 years with a minimum age of 18 years and maximum age of 68 years. Men made up about 80% of the study's participants and the remaining were females. Most of the individuals were diagnosed with C1 type of fracture according to AO/OTA classification. About 52%, 26%, and 22% of study participants had a C1, C2, or C3 type of fracture, respectively. Most of the individuals had a right-side injury (65%). Among the study participants, about 75% had fractures due to road traffic accidents (RTAs). The general characteristics of the study participants are presented in Table 1.

**Table 1:** General characteristics of the study participants (n=30)

S.no	Variables		Frequency	Percentage
1	Age in years: mean: 48 years; standard deviation: 13.25 years			
2	Gender	Male	24	80
		Female	6	20
3	Types of fracture	C1	16	52
		C2	8	26
		C3	6	22
4	Side of involvement	Right side	20	65
		Left side	10	35
5	Mechanism of injury	Road traffic accident	23	75
		Accidental fall	7	25

Common complications observed during the plating procedure among the study participants were excessive bleeding, difficulty in reduction, superficial infections, and knee stiffness. The prevalence of these complications is shown in Table 2.

**Table 2:** Summary of complications faced during locking compression plating procedure (n=30)

S.no	Complications faced during the procedure		Frequency	Percentage
1	Yes	Excessive bleeding	3	10
		Difficulty in reduction	2	7
		Superficial infection	1	4
		Knee stiffness	1	4
2	No		23	75

The mean time for the radiological union was 15 weeks with a standard deviation of 1.06 weeks. In this study, there were no instances of infection or angular deformity greater than 5 degrees. About 80% of the study participants had a range of motion (ROM) above 120 degrees at the end of the six-month follow-up period. About 4.5% of the participants had less than 90 degrees of ROM (Table 3). The rate of favorable functional outcomes in the study participants at the end of six months was 94.5%.

**Table 3:** Distribution of the study participants according to their functional outcome (n=30)

S.no	Degree of range of motion	Frequency	Percentage
1	More than 120 degrees	24	80
2	90-120 degrees	4	14.5
3	Less than 90 degrees	2	4.5

As for complications, non-union affected about 2% of study participants. About 4.5% of the study participants had severely restricted ROM. The complications from the procedure are presented in Table 4.

**Table 4:** Summary of complications among the study participants after the procedure (n=30) ROM: range of motion

S.no	Complications		Frequency	Percentage
1	Yes	Angular deformity	0	0
		Infections	0	0
		Non-union	1	2
		Severe restriction of ROM	2	4.5
2	No		27	93.5

According to Neer's score for straight leg raises among the study participants, about 12 cases had an excellent score (40%), 15 cases (50%) had a satisfactory score, two cases (6.7%) had an unsatisfactory score, and only one case had a poor score. According to Neer's score, about 94.5% of the study participants had good functional outcomes (Table 5).

**Table 5:** Distribution of the study participants according to their Neer’s score category (n=30)

S.no	Neer’s score for straight leg rises	Frequency	Percentage
1	Excellent	12	40
2	Satisfactory	15	50
3	Unsatisfactory	2	6.7
4	Poor	1	3.3

## Discussion

Throughout the history of fracture treatments, distal femur fracture has presented significant therapeutic challenges. The majority of these surgical failures were brought on by insufficient fracture fragment fixation. Age, intra-articular involvement, and the amount of joint movement are prognostic factors for distal femur fracture.

The majority of the patients (80%) in our study were men, with the remaining 20% being women. This gender disparity may be attributed to the fact that men engage in more outdoor activities than women, as reported in a study by Ru et al. [13]. This supports Link and Babst's claim that fractures occur in a bimodal distribution. That is, one group was made up primarily of males where the major cause is injury and the other group was made up of older females, in whom the major cause was osteoporosis [14]. Another study by Khadem et al. in 2022 also had a similar finding: about 80% of the study participants were men [15]. In contrast to the above results, a study by Kiyono et al. in 2019 in Japan found that 67% of the study participants having distal femur fractures were females [16]. This difference may be due to differences in study settings, yet it needs further exploration.

The current study's participants ranged in age from 18 to 68 years, with 48 years being the mean age. This is in line with a study by Trivedi et al., who found that the majority of distal femoral fractures were found in people over 50 years of age. The osteoporosis in this group could make fixation difficult [17]. Also, in a study by Kiyono et al., the mean age of the participants was 68 years [16]. The study by Harvin et al. in 2017 had similar findings. They found that the mean age of the study participants was 60 years [18]. This indicates that the risk of getting distal femoral fractures increases with age.

In our study, approximately 20 (65%) patients had right-sided fractures, while the remaining 12 patients had left-sided fractures. There were no patients with bilateral femur fractures. This contrasts with the studies by Ru et al. and Daroch et al., where the left leg was more commonly involved. This difference in laterality may result from variations in genetic susceptibility, the prevalence of other risk factors like injury mechanisms and osteoporosis, or both [13,19].

In the current study, the mechanism of injury in 75% (n=23) of the cases was determined to be an RTA, while the remaining 25% of the cases reported an unintentional fall. This finding is in line with those of the related studies conducted by Daroch et al., Link et al., and Ru et al. [13,14,19].

In this study, 25% of patients experienced intraoperative complications, which included excessive bleeding in 10% (n=3) and difficulty in reduction in 7% of cases. Patients who experienced excessive bleeding typically lost 550-650 mm of blood, while in other cases the blood loss was 450 mm. Similarly, a study by Ru et al. found these to be among the most frequent intraoperative complications [13].

In the current study, 65% of the patients were recorded as having achieved union at three months, with the remaining patients achieving it at six months. The outcomes matched the findings of Daroch et al.'s study [19]. With one exception, the radiological union was achieved in all cases. Only one case (2%) involved non-union. In the current study, the mean time for the radiological union was 15 weeks. The patient was instructed to begin partial weight bearing when the callus formation began. In a study by Kanabar et al., the time for union ranged from 12 to 19 weeks [20]. Similar results were found in a study done in 2021 by Park et al., where the mean time for the union was 18.4 weeks [21]. Another study by Kim et al. in 2017 revealed that the mean time for the union was 17.1 weeks [22].

Of note, 24 cases (80%) had full flexion achieved at or above 120 degrees, while four patients (14.5%) had flexion between 90 and 120 degrees and the remaining two patients had flexion below 90 degrees. The current study's ROM was comparable to that of Rademaker et al.'s study, which was based on Neer's criteria [23].

Neer's criteria were used to determine the knee score. Out of 30 cases, 12 cases (40%) had Neer's scores of 85 or higher and were considered to have excellent outcomes; 15 cases (50%) had scores of 75-85 and were considered to have satisfactory outcomes; two cases (6.7%) had scores of 55-74 and were considered unsatisfactory, and only one case (or 3.3%) had a score of 55 and was considered poor; 84% of the 67 patients in the study by Rademaker et al. had results that were satisfactory to excellent [23]. Daroch et al. found that 83.34% of the results from their study of 30 patients were satisfactory or excellent, and numerous other researchers have also found similar results [19]. The current study found that 85.1% of patients had results that ranged from excellent to satisfactory.

Limitations of the study

We believe that a multi-centric study with a larger sample size can produce better results, even though the current study met the minimum sample size requirement. Only those patients with OA/OTA type C fractures were included in this study. The effectiveness of LCP in patients with other types of fractures needs to be investigated further. Our study's follow-up period was very brief. Further research is required to understand the functional outcomes of this procedure in the long term.

## Conclusions

Based on our findings, the risk of distal femur fracture is higher among men. RTAs are still the most common cause of distal femur fractures. Union of fracture occurs at 15 weeks after management with LCP. For patients with distal femoral fractures, the LCP treatment leads to good functional and radiological outcomes.
